# Maximum preimplantation monosyllabic score as predictor of cochlear implant outcome

**DOI:** 10.1007/s00106-019-0648-0

**Published:** 2019-04-03

**Authors:** Ulrich Hoppe, Thomas Hocke, Anne Hast, Heinrich Iro

**Affiliations:** 1 Audiologische Abteilung, Hals-Nasen-Ohrenklinik, Kopf- und Halschirurgie, Universitätsklinikum Erlangen, Waldstr. 1, 91054 Erlangen, Germany; 2 Cochlear Deutschland GmbH & Co. KG, Hanover, Germany

**Keywords:** Cochlear implants, Speech audiometry, Hearing tests, Speech discrimination tests, Hearing loss

## Abstract

**Objective:**

This study investigated the speech perception of cochlear implant (CI) recipients with measurable preoperative ipsilateral speech perception. These data should support improved individual counselling of CI candidates.

**Materials and methods:**

Pre- and postoperative speech audiometric parameters were analyzed, including maximum score for phonemically balanced words (PB_max_) and monosyllabic score at a normal conversational level of 65 dB_SPL_, with hearing aids one hand and CI on the other. Data of 284 experienced adult CI wearers were grouped and evaluated in terms of preoperative PB_max_.

**Results:**

The preoperative PB_max_ was exceeded by the postoperative monosyllabic score in 96% of cases. The overall median postoperative score was 72.5%. The groups with preoperative PB_max_ > 0% showed significantly better speech perception scores with CI than the group with PB_max_ = 0%. Median improvement compared to the preoperative monosyllabic score with hearing aids was 65 percentage points, independent of preoperative PB_max_.

**Conclusion:**

The preoperatively measured PB_max_ may be used as a predictor for the minimum speech perception obtained with CI. This is of high clinical relevance for CI candidates with a PB_max_ above zero.

## Background

Cochlear implantation is an established treatment for patients with severe-to-profound hearing loss up to total deafness [20]. In the early years of treatment, only patients with functional deafness and no speech perception with acoustic amplification were considered as candidates for a cochlear implant (CI). In the past two decades, the audiological indication criteria have been widened considerably [4, 11]. Today, some candidates still have substantial residual hearing on the side to receive the CI. Concerning the contralateral side, hearing-impaired patients with all degrees of hearing loss down to normal hearing have successfully received an implant [1, 16, 20]. The reasons for this were the continuous improvement of CI treatment in surgery [19, 20], technology [2, 7, 8, 17, 28], and rehabilitation [26, 29]. Furthermore, an increasing number of subjects with significant preoperative ipsilateral hearing have been considered for cochlear implantation [27]. For these candidates in particular, the individual prediction of postoperative speech perception with respect to the preoperative assessment is an absolute clinical necessity, as cochlear implantation may impair residual hearing [19, 20]. Various studies have been performed to investigate the factors influencing postoperative speech perception in large recipient groups [2, 8, 18, 29].

Blamey et al. [2], reporting on 2251 recipients, identified five main factors influencing postoperative speech perception scores in different ways: duration of severe-to-profound hearing loss, age at implantation, age at onset of severe-to-profound hearing loss, etiology, and duration of implant experience. The relation between preoperative and postoperative speech recognition was not discussed explicitly. This was presumably because of the multicenter and multilingual study design, which introduced inherent limitations for comparing speech scores before and after cochlear implantation. Closer examination of their data reveals a further limitation: Only a small proportion of recipients had preoperative monosyllabic scores of more than 0%.

Holden et al. [8], reporting on 114 subjects, found a correlation between preoperative sentence recognition score and postoperative monosyllabic score. However, as with the study of Blamey et al., the mean preoperative sentence recognition scores were rather low, with most of the subjects scoring close to or exactly 0% with a mean of 16.4% ± 18%. In a multicenter study, Gifford et al. [4] compared preoperative monosyllabic word (consonant—nuclear vowel—consonant, CNC) scores of 22 subjects in the best-aided condition with their postoperative CI-only and, if possible, bimodal scores. Their results, together with those of Holden et al., suggest that the better the preoperative speech recognition ability, the better the CI score. This finding has since been confirmed by various studies [3, 14, 18].

### Preoperative speech perception

For hearing aid (HA) and CI evaluation in German-speaking countries, monosyllabic and sentence tests are mostly used [15]. The Freiburg monosyllabic test plays a specific role; it is conducted with headphones within the standardized speech audiogram as well as in the free-field situation with HA or CI. This yields information about speech intelligibility at conversation levels and close to the discomfort level [15, 25].

Speech perception measures used in preoperative evaluations include the score for recognition of phonemically balanced monosyllabic words at conversation level of 65 dB with a hearing aid, Word Recognition Score (WRS)_65_(HA); another is the maximum recognition score for phonemically balanced monosyllabic words (WRS_max_; also often referred to as PB_max_). The latter is measured as a part of the performance-intensity function by using air-conduction headphones. The presentation level for WRS_max_ may vary between individuals, and—especially for higher degrees of hearing loss—it can reach values slightly below the level of discomfort [5].

When evaluated together with the pure-tone audiogram, WRS_max_ allows for an initial assessment of the best speech recognition that can be achieved with acoustic amplification [10, 24]. For most individuals, WRS_max_ is higher than WRS_65_(HA) [12, 13]. Halpin and Rauch [6] discussed WRS_max_ in connection with the information-carrying capacity (ICC) of the auditory system. The WRS_max_ can be regarded as an estimator for the ICC. Halpin and Rauch emphasized that similar pure-tone audiograms may lead to different speech perception abilities. The pure-tone audiogram captures the attenuation component of hearing loss; other potential impacts of a cochlear hearing disorder, such as reduced temporal or spectral resolution, are not assessed. In addition to the pure-tone audiogram, WRS_max_ captures implicitly the impact of the reduced temporal and spectral resolution of the entire auditory system.

Recent studies [10, 11, 15, 21–23] of hearing-aid users have reported a considerable proportion of users, even among those with moderate hearing loss, who were unable to convert their ICC (measured as WRS_max_) into aided speech perception at conversation levels. This mismatch can be explained, at least in users with higher degrees of hearing loss: WRS_max_ is measured near the discomfort level [10]. The insufficient dynamic range [30] of that group of hearing-aid users, together with their intolerance of the high acoustic amplification needed, limits the potential benefit of hearing-aid provision in those cases.

The aim of this retrospective study was to investigate speech perception following cochlear implantation in subjects who had demonstrated substantial ICC as measured by a preoperative WRS_max_ above 0%. Therefore, speech perception scores of recipients with different levels of preoperative monosyllabic scores were compared. Furthermore, the value of WRS_max_ as a predictive factor for postoperative speech perception scores was assessed.

## Methods

### Patients

A total of 550 patients had received a Nucleus cochlear implant (Cochlear Ltd, Sydney, Australia) in the ENT department of the University Hospital of Erlangen between January 2010 and June 2014; all of these patients’ files were reviewed. After excluding pediatric implantations, there were 312 adult subjects, each of whom had received a CI, either a Nucleus CI24RE(CA) (*N* = 208) or Nucleus CI512 (*N* = 104); these models have identical perimodiolar electrode arrays and function, but different receiver/stimulator housings. The implantation was carried out by cochleostomy (*N* = 81), by round-window insertion (*N* = 41), or by round-window enlargement (*N* = 190). Correct intracochlear electrode positioning was verified by postoperative imaging using either conventional X‑ray or computer-aided tomography.

Of these 312 adult cases, 28 were excluded from further evaluation for surgical and other reasons, specifically:

- Prelingual deafness (11)
- Mother tongue not German (8)
- Change of rehabilitation center (2)
- Meningioma (1)
- Incomplete insertion (3)
- Tip fold-over (1)
- Severe mental retardation (1)
- No preoperative hearing aid experience, owing to atresia (1)

The cases were grouped according to their preoperative WRS_max_ score into three groups: Group 1 consisted of cases with a WRS_max_ of 0, group 2 had a WRS_max_ above 0 and up to 50% (inclusive), and group 3 had a WRS_max_ above 50%. Table 1 summarizes the statistical data for age and preoperative speech perception measures.

**Table 1:** Group demographic information including, age, preoperative WRS_max_ (headphones), and the preoperative aided WRS_65_(HA)

		Group 1 *N* = 121	Group 2 *N* = 126	Group 3 *N* = 37
Age at implantation (years)	Minimum	19	21	22
	1st quartile	45	53	45
	*Median*	*61*	*65*	*64*
	3rd quartile	70	72	72
	Maximum	85	92	78
WRS_max_ (%)	Minimum	0	5	55
	1st quartile	0	15	60
	*Median*	*0*	*25*	*70*
	3rd quartile	0	35	75
	Maximum	0	50	90
Preoperative aided WRS_65_(HA) (%)	Minimum	0	0	0
	1st quartile	0	0	0
	*Median*	*0*	*0*	*15*
	3rd quartile	0	15	35
	Maximum	25^a^	50	55

*WRS*_*max*_ maximum Word Recognition Score, *WRS*_*65*_*(HA)* Word Recognition Score at 65 dB with hearing aid

^a^One patient had a WRS_65_(HA) = 25% and WRS_max_(headphones) = 0%; all other patients in group 1 had a WRS_65_(HA) = 0%

### Preoperative speech audiometry

Apart from WRS_max_, which was measured by headphone, aided monaural monosyllable perception was measured in free field in a 6 × 6-m anechoic booth at 65 dB, WRS_65_(HA). The loudspeaker was placed 1.5 m in front of the patient (0° azimuth). The contralateral ear was masked appropriately with wideband noise presented through headphones (DT48; beyerdynamic, Heilbronn, Germany). All CI candidates had at least 3 months of HA experience. The last fitting process had been within the 3 months before audiometric assessment. Before measurements, HA function was checked technically by hearing-aid acousticians in the ENT department. In addition to the visual inspection and feedback provocation, it was ensured that the prescribed hearing aids provided sufficient amplification, corresponding to the individual’s hearing loss. With regard to the fitting, in cases where any problems were encountered, coupler or in situ measurements were performed in order to ensure sufficient acoustic amplification.

### Postoperative speech audiometry

The postoperative score with a CI for the Freiburg monosyllabic test at 65 dB sound pressure level (SPL), WRS_65_(CI), was measured 6 months after CI activation. The same audiometric setup as for the preoperative WRS_65_(HA) was used, including contralateral masking.

### Data analysis

The Matlab® Software R2013a (MathWorks, Natick, MA, USA) was used for performing calculations and producing figures. Since the speech perception scores were not normally distributed (*p* < 10^−6^ by the Shapiro–Wilk test) nonparametric analysis was performed. Group comparisons were analyzed with the Kruskal–Wallis test in combination with post hoc analysis. Individual pretest–posttest comparisons of speech perception scores were undertaken according to Holube et al. [9]. Correlation analysis was performed using the Spearman rank correlation.

## Results

**Fig. 1:**
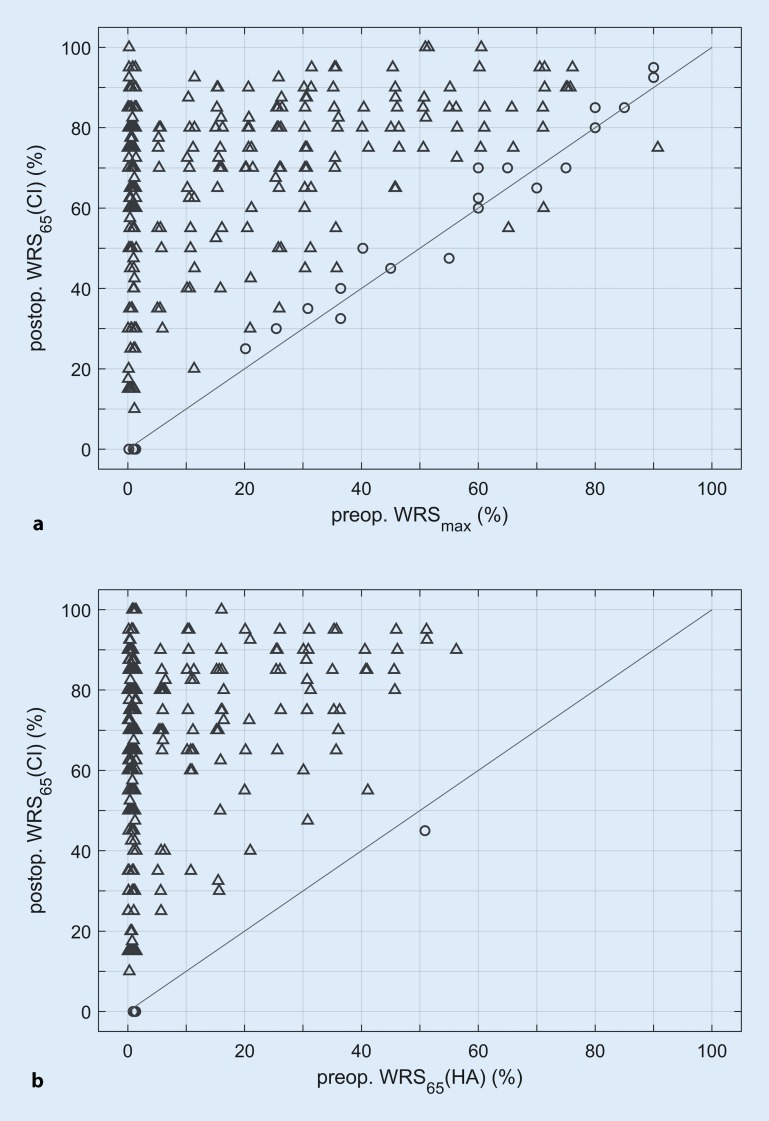
Preoperative (*preop.*) and 6‑month postoperative (*postop.*) speech perception. **a** Maximum Word Recognition Score, *WRS*_*max*_, measured preoperatively with headphones in the ear to receive a cochlear implant (*CI*), and postimplantation monosyllabic recognition score at 65 dB_SPL_, WRS_65_(CI), in free field. **b** Preoperative monaurally aided monosyllabic recognition score at 65 dB_SPL_ (sound pressure level, SPL), WRS_65_(HA), in free field and postimplantation WRS_65_(CI). *Triangles* represent cases with significant difference and *circles* represent those with no significant difference between pre- and postoperative findings. *HA* hearing aid

The scatter plot in Fig. 1 shows the relationship between the postoperative WRS_65_(CI) (*y*-axis) and the preoperative WRS_max_ (Fig. 1a) or WRS_65_(HA) (Fig. 1b). Points above the diagonals represent higher postoperative scores, and points below them represent lower ones. In Fig. 1a, points cover the entire area above the diagonal. The triangles denote significant changes at the individual level (as defined in [9]).

Analysis of the correlation between preoperative WRS_max_ and postoperative WRS_65_(CI) was performed for all patients in groups 2 and 3. The rank correlation coefficient is *r* = 0.39 with *p* = 3.4 × 10^−7^. The majority (156; 96%) of the 163 CI recipients in groups 2 and 3 with a WRS_max_ above 0% had postoperative WRS_65_(CI) scores that were equivalent to or surpassed their preoperative WRS_max_. However, seven recipients failed to achieve their preoperative WRS_max_. Their audiometric data are displayed in Table 2. However, these subjects showed improved speech perception with the CI, compared with the HA score, at 65 dB_SPL_. The scatter plot in Fig. 1b shows 98% of the points above the diagonal, indicating improved speech perception at conversation level after 6 months of CI experience.

**Table 2:** Audiometric data of the seven subjects with substantial preoperative hearing who failed to achieve at least the preoperative WRS_max_ with CI

Age (years)	4FPTA (dB)	WRS_max_ (%)	WRS_max_ Level (dB)	WRS_65_(HA) (%)	WRS_65_(CI) (%)	WRS_65_(CI)—WRS_max_ (% points)
65	88	90	120	0	75	−15*
77	71	65	110	0	55	−10*
76	74	70	110	0	60	−10*
68	83	55	110	30	47.5	−7.5
74	76	75	110	0	70	−5
78	83	70	110	35	65	−5
64	82	35	110	15	32.5	−2.5

*4FPTA* four-frequency pure tone average, *WRS*_*max*_ maximum Word Recognition Score, *WRS*_*65*_*(HA)* Word Recognition Score at 65 dB with hearing aid, *WRS*_*65*_*(CI) *Word Recognition Score at 65 dB with cochlear implant

*Significant differences according to Holube et al. [9]

**Fig. 2:**
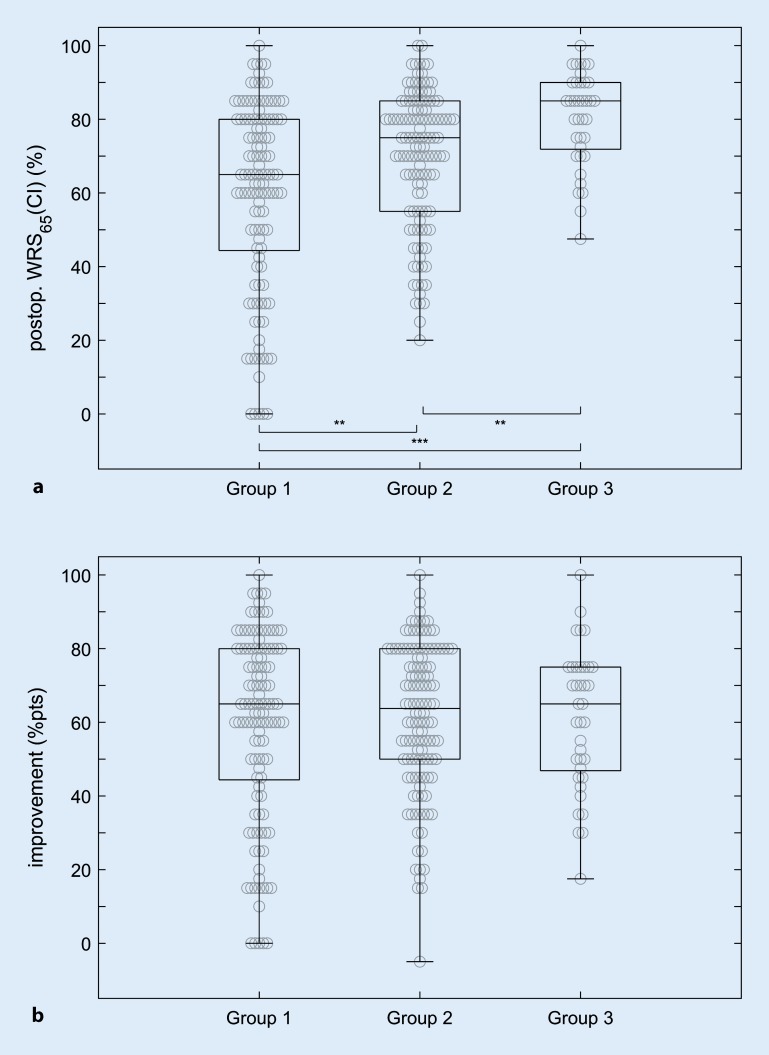
Box plots of the postoperative (*postop*.) speech recognition scores for the three groups. **a** Word Recognition Score (WRS) with cochlear implant (CI) at 65 dB_SPL_ (sound pressure level, SPL), WRS_65_(CI), in free field. **b** Improvement, i. e., the difference between the preoperative (*preop*.) monaural monosyllabic score with hearing aid at 65 dB, WRS_65_(HA), and WRS_65_(CI): WRS_65_(CI)—WRS_65_(HA). *Box plots* median, first, and third quartile, minimum, and maximum. *Asterisks* significance levels as found in post hoc analysis: *p < 0.05, **p < 0.01, ***p < 0.001

Figure 2a shows the distribution of WRS_65_(CI) for the three groups as box plots. The median WRS_65_(CI) is 65%, 75%, and 85% for groups 1, 2, and 3, respectively. According to the Kruskal–Wallis test, postoperative WRS_65_(CI) differed significantly between the three groups, H(2) = 26.2, *p* < 0.001. Pairwise post hoc comparisons with adjusted *p* values showed that the median WRS_65_(CI) differed for all three groups (*p* < 0.01). Analysis of these differences for the groups did not reveal any statistically significant difference between the median values, H(2) = 0.105, *p* = 0.95. This means that all patients experienced a comparable improvement of around 65 percentage points at conversation level, independently of their preoperative WRS_max_.

**Fig. 3:**
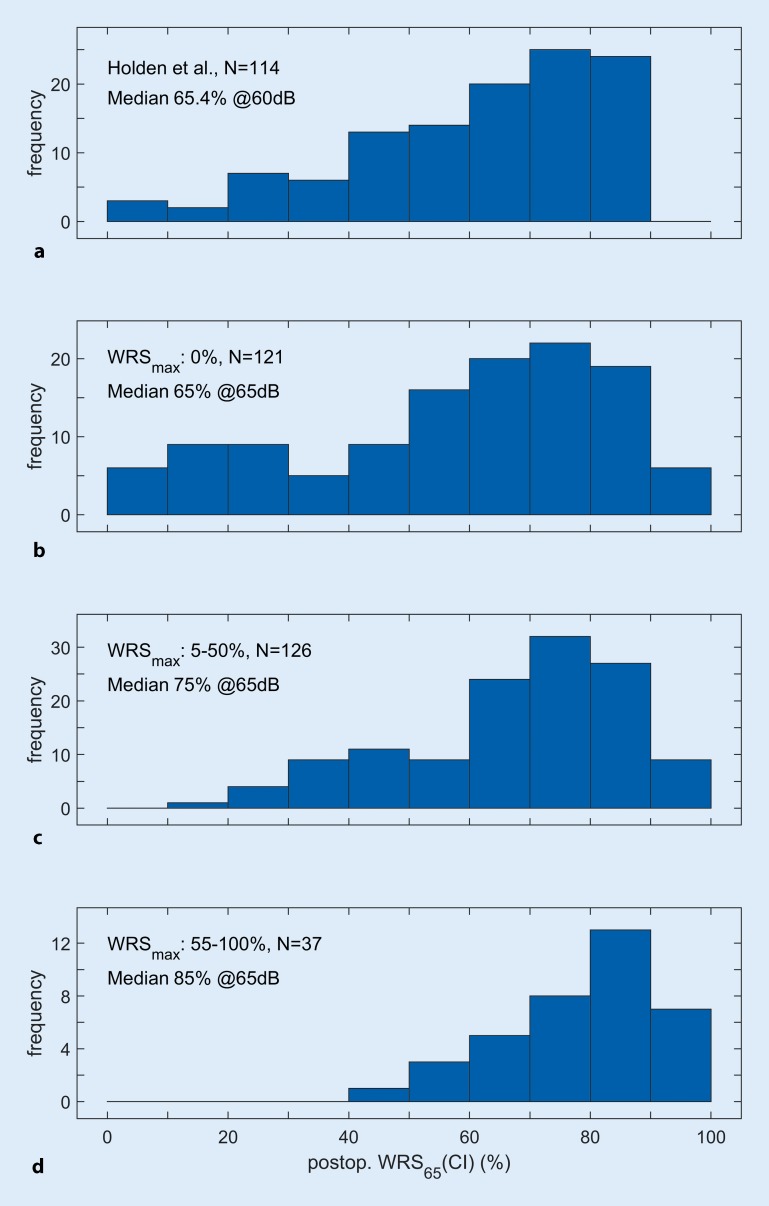
Histograms of postoperative (*postop.*) recognition scores. **a** Final CNC scores of 114 postlingually deafened subjects as reported by Holden et al. [8]. **b** Six-month postoperative monosyllabic score of the recipients with no preoperative (*preop.*) monosyllabic recognition ability (Table 1, group 1, WRS_max_ = 0%). **c,** **d** Data for recipients with measurable preoperative speech recognition (Table 1, group 2 and 3): **c** WRS_max_ ≤ 50%, **d** WRS_max_ > 50%. *WRS*_*max*_ maximum Word Recognition Score, *CI* cochlear implant

The histograms in Fig. 3 show the postoperative monosyllabic score with CI for groups 1–3 (Fig. 3b–d). It is evident that the distribution character differs among the groups. For group 1 the distribution shows two peaks (Fig. 3b). Figure 3a shows corresponding results from Holden et al. [8] for the CNC score of 114 postlingually deafened adults, measured 24 months postoperatively at 60 dB_SPL_. It is clear that the distribution of speech perception scores found by Holden et al. is most closely comparable to that of our group 1 (Fig. 3b).

## Discussion

With a view to supporting the audiological part of the indication and individual counselling process for CI candidates, we investigated the predictive value of the preoperative maximum speech recognition score.

For the CI recipients in groups 2 and 3 with a preoperative WRS_max_ above 0%, we found a significantly higher postoperative monosyllabic score than for the recipients in group 1 with WRS_max_ = 0%. For patients with WRS_max_ > 0%, the postoperative monosyllabic score with CI was significantly correlated with the preoperative WRS_max_. This correlation reinforces the interpretation of WRS_max_ as a measure of ICC [6]. The ICC is limited by sensorineural pathologies. Since the WRS_max_ is measured considerably above the individual’s hearing threshold, it reflects, more closely than other audiometric measures do, the individual’s neuronal processing capacity. For 96% of the cases with a WRS_max_ above 0%, we found a postoperative monosyllabic score WRS_65_(CI) equal to or above the preoperative WRS_max_. Consequently, the preoperative WRS_max_ can be interpreted as a lower limit (minimum predictor) for speech perception with CI after 6 months.

### Estimation of speech perception with CI

For CI candidates with residual speech perception there is a residual risk of postoperatively decreased speech perception, even under optimum conditions [19]. Therefore, the individual prognosis of postoperative speech perception is of special importance for patients in groups 2 and 3. Almost all patients with preoperative WRS_max_ > 0 had a WRS_65_(CI) that surpassed, or at least equaled, their preoperative WRS_max_. An advantage of the reference to the WRS_max_ and not to the WRS_65_(HA) is the distribution of the data: 60% of the CI candidates attained a preoperative WRS_max_ above 0%, whereas only 32% scored an WRS_65_(HA) above 0%. Additionally, WRS_max_ covers a range from 0 to 90%, allowing for a more finely differentiated description of the candidates’ speech perception capabilities than does the WRS_65_(HA), with a range from 0 to only 55%.

### Patients without preoperative monosyllabic speech perception

Inherently, WRS_max_ cannot provide additional information on the postoperative speech perception of persons in group 1 (for whom WRS_max_ = 0%). However, this has little influence on the clinical decision, owing to the lack of alternative therapies. The postoperative speech perception scores of group 1 showed a large variability (Fig. 3b). This finding is in line with the results of other groups [2, 8, 17, 29]. A detailed comparison of the results of Holden et al. ([8]; Fig. 3a) with those of this patient group without preoperative speech perception (WRS_max_ = 0%; Fig. 3b) shows a similar distribution of monosyllabic test scores with CI, despite the different examination conditions (6 vs. 24 months, 65 vs. 60 dB, Freiburg test vs. CNC). In group 1 it must be expected that a certain proportion (about 4%) of recipients will not develop monosyllable discrimination during postoperative development. Studies by Blamey et al. [2] indicate a similar range (3–4%) for this proportion.

### Patients with preoperative speech perception

The postoperative speech perception was significantly higher for the two groups with preoperative monosyllabic speech perception greater than zero (WRS_max_ > 0%) than for group 1. Therefore, our results support the current trend toward the treatment of patients with substantial speech perception [20]. The improvement in speech perception with CI, by 65 percentage points, was equal for all three groups. This means that better speech perception with CI was associated with better preoperative WRS_max_. This also supports a posteriori the provision of CIs to patients with high preoperative WRS_max_, particularly in cases in which the maximum monosyllabic test score is far above the speech perception achieved with HAs at conversational level. In the present study this was the case for all patients with high WRS_max_.

### Speech perception with CI in long-term development

Even though this study did not explicitly address the postoperative development of the WRS_65_(CI), this aspect did influence the study design. Thus, Krüger et al. [17] reported an initially steeper growth of the WRS_65_(CI) with current CI systems over time than observed earlier in study populations. Furthermore, the results of Holden et al. [8] show that 90% of the final (i. e., after 2 years) monosyllabic test score was already reached after 6 months. For this reason, we investigated the correlation between the WRS_65_(CI) after 6 months and the preoperative WRS_max_ in order further to minimize the variability. Future studies may investigate the influence of rehabilitation, motivation, communicative environment, and additional training measures [26, 29]. These variables are difficult to control for large patient groups and were therefore not taken into account in this study.

This methodological consideration leads to the observation that speech perception scores may increase during long-term postoperative development, which in turn strengthens the potential of the WRS_max_ as a predictor of the minimum expected result. This affects the single case (Fig. 1b) where the postoperative WRS_65_(CI) was lower (by 5 percentage points) than the preoperative WRS_65_(HG). Here, a WRS_65_(CI) of 80% was achieved after 12 months. The incidence of such cases [19] emphasizes the need for a conservative minimum predictor.

## Practical conclusion

- The WRS_max_ is a useful measure that may offer substantial support for *individual* CI counselling and treatment decisions.
- The preoperative maximum monosyllabic word perception, WRS_max_, can predict the minimum postoperative speech perception with a reliability of 96%.
- Better preoperative speech perception yields better speech perception with a CI.
- In the patient group with a preoperative monosyllabic score above 0%, all CI recipients had at least some postoperative monosyllabic perception.
- The median improvement following CI provision was 65 percentage points.
